# Characterizing hospitalization trajectories in the high-need, high-cost population using electronic health record data

**DOI:** 10.1093/haschl/qxad077

**Published:** 2023-12-06

**Authors:** Scott S Lee, Benjamin French, Francis Balucan, Michael D McCann, Eduard E Vasilevskis

**Affiliations:** Section of Hospital Medicine, Department of Medicine, Vanderbilt University Medical Center, Nashville, TN 37203, United States; Department of Biostatistics, Vanderbilt University Medical Center, Nashville, TN 37203, United States; Section of Hospital Medicine, Department of Medicine, Vanderbilt University Medical Center, Nashville, TN 37203, United States; Section of Hospital Medicine, Department of Medicine, Vanderbilt University Medical Center, Nashville, TN 37203, United States; Division of Hospital Medicine, Department of Medicine, School of Medicine and Public Health, University of Wisconsin, Madison, WI 53726, United States

**Keywords:** high utilizers, hospital utilization, electronic health record data, growth mixture modeling

## Abstract

High utilization by a minority of patients accounts for a large share of health care costs, but the dynamics of this utilization remain poorly understood. We sought to characterize longitudinal trajectories of hospitalization among adult patients at an academic medical center from 2017 to 2023. Among 3404 patients meeting eligibility criteria, following an initial “rising-risk” period of 3 hospitalizations in 6 months, growth mixture modeling discerned 4 clusters of subsequent hospitalization trajectories: no further utilization, low chronic utilization, persistently high utilization with a slow rate of increase, and persistently high utilization with a fast rate of increase. Baseline factors associated with higher-order hospitalization trajectories included admission to a nonsurgical service, full code status, intensive care unit-level care, opioid administration, discharge home, and comorbid cardiovascular disease, end-stage kidney or liver disease, or cancer. Characterizing hospitalization trajectories and their correlates in this manner lays groundwork for early identification of those most likely to become high-need, high-cost patients.

## Introduction

High-need, high-cost (HNHC) patients represent a distinct challenge for the US health care system. On one hand, these patients account for a disproportionate share of health care utilization and costs, making them a natural target for intervention.^1,2^ On the other hand, despite constituting a small minority of the total US population, HNHC patients are extremely diverse,^3^ such that one-size-fits-all approaches to addressing their needs are unlikely to succeed.

Recent research has attempted to unravel this heterogeneity, using administrative and claims data to identify distinct clusters within the HNHC population with respect to sociodemographic and clinical characteristics.^4-10^ However, despite these advances, significant gaps persist in our understanding of (1) how high utilization first emerges and (2) why some patients become persistently high utilizers, whereas others’ utilization is high only transiently.^2^ These gaps highlight the importance of focusing not only on high utilization in a cross-sectional, aggregate sense (eg, total hospitalizations in a given year) but also in terms of longitudinal patterns, or trajectories, of utilization and risk that evolve dynamically over time, as has recently been studied in other contexts.^6,11-16^ Mapping these trajectories provides the groundwork for early identification of patients most likely to have high utilization.

Against this backdrop, we sought to characterize hospitalization trajectories by applying advanced statistical modeling techniques to longitudinal electronic health record (EHR) data. We posited the following hypotheses:

- Hypothesis 1: Distinct clusters within the HNHC population can be identified with respect to longitudinal hospitalization trajectories.
- Hypothesis 2: These trajectory clusters can be differentiated based on baseline patient and hospitalization characteristics.

Raw EHR data pose challenges for evaluating these hypotheses, primarily because any chronological interval (eg, a given calendar year) inevitably captures patients at different stages of their trajectories. To overcome this challenge, we introduce the concept of *utilization episodes*. As defined in this study, a utilization episode has 3 stages (Figure S1): (1) an initial period of low utilization, followed by (2) a rising-risk period of higher utilization, and finally, (3) the utilization trajectory proper. In this way, utilization episodes establish a common starting point for a cohort of patients whose utilization can then be compared longitudinally. Ensuring the presence of the first 2 stages allows us to observe “incident” high utilization—ie, high utilization as it emerges and evolves over time.

We further introduce the concept of the *index hospitalization* as the starting point of the utilization trajectory. The intuition for this approach is based on a clinical scenario common to programs and research studies for the HNHC population. Typically, such programs aim to enroll patients in the early stages of high utilization. While this has the potential to prevent unnecessary utilization, it also runs the risk of targeting patients who may turn out not to be high utilizers. As such, predicting a utilization trajectory based on information available at the rising-risk stage has high clinical and policy importance.

Bringing these concepts together, we sought to (1) construct hospital utilization episodes among a cohort of rising-risk patients, (2) characterize clusters of hospitalization trajectories after the rising-risk period, and (3) identify correlates of hospitalization trajectories based on data available at the time of the index hospitalization.

## Data and methods

### Clinical setting and data source

This study took place at Vanderbilt University Medical Center (VUMC), an academic medical center comprising 1600 beds across 6 acute-care hospitals in middle Tennessee. In November 2017, VUMC transitioned its EHR to Epic, a proprietary software developed by Epic Systems (Verona, Wisconsin). VUMC's Epic-based clinical database served as the data source for this study.

**Figure 1.**
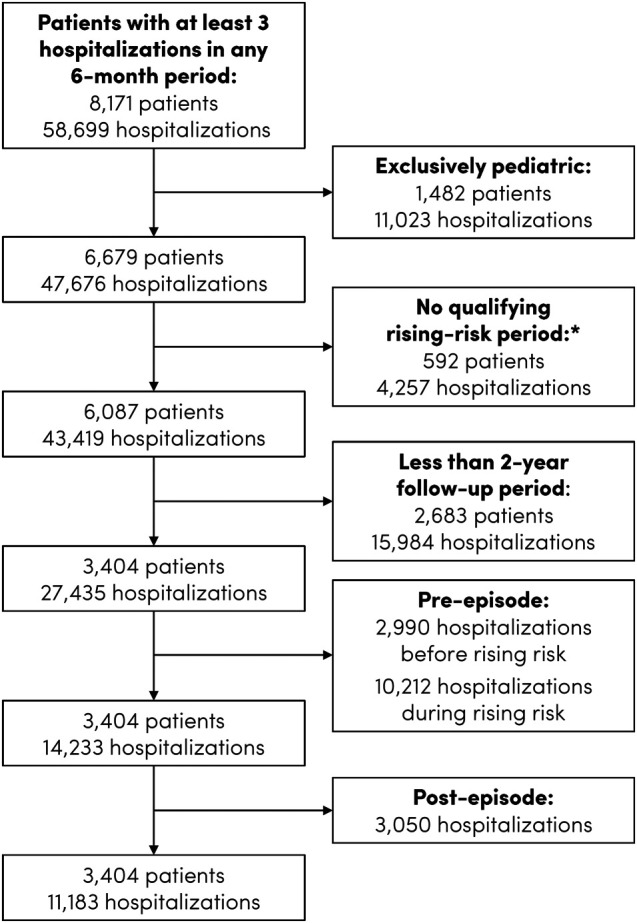
Flow diagram for inclusion in study. *First instance of 3 hospitalizations in a 6-month period with no more than 2 hospitalizations in the preceding 6-month period.

The dataset constructed for this study spanned the period from November 1, 2017 (the launch date of the EHR software) to July 6, 2023, for a total follow-up period of 5.7 years. Because ethical and computational constraints prohibited extracting all data for all patients, as an initial screen, patients with at least 3 hospitalizations in any 6-month period were extracted from the source database (Figure 1). Hospitalizations included both acute (ie, starting in the emergency department [ED]) and elective admissions. Exclusively pediatric patients—ie, patients for whom all hospitalizations occurred before age 18—were excluded.

### Construction of utilization episodes

To construct each utilization episode (Figure S1), the first step entailed identifying a qualifying rising-risk period, which was defined as the first instance of (1) 3 hospitalizations in a 6-month period with (2) no more than 2 hospitalizations in the preceding 6-month period. Patients who did not meet this case definition, including those with a preceding low-risk period shorter than 6 months, were excluded.

The third hospitalization in the rising-risk period was deemed the index hospitalization, and its start date marked the beginning of the hospitalization trajectory. The duration of the follow-up period was set at 24 months (720 days). To be included, patients must have been at risk for hospitalization during the entire follow-up period (ie, the index hospitalization must have occurred at least 24 months before the end date of the dataset) or died during the period.

For each hospitalization, data included hospitalization characteristics (eg, location, admission date, length of stay, primary team), patient characteristics (eg, demographics, insurance, chronic comorbidities present at the time of hospitalization), and all medications administered during the hospitalization. Chronic comorbidities, including medical, psychiatric, pain, substance use, and social determinants of health (SDOH) diagnoses, were drawn from the “problem list,” a feature of the Epic EHR in which clinicians maintain a running list of *International Classification of Diseases, 10th Revision* (ICD-10)–coded problems and diagnoses for a given patient.

### Statistical methods

#### Hypothesis 1: identification of hospitalization trajectory clusters

To characterize patterns of longitudinal trajectories of hospitalization among rising-risk patients, we used growth mixture modeling (GMM), a statistical technique for identifying latent classes/clusters of growth trajectories that follow similar patterns over time.^17^ We modeled cumulative hospitalizations as a linear-quadratic function of time, thereby allowing for curvilinear trajectories. Patient-specific random intercepts and slopes for time were used to allow for heterogeneity in trajectories across patients. In total, we tested 5 growth mixture models in which the cluster count varied from 1 to 5. For each model, we calculated standard fit statistics and chose the model best representing the “elbow” of the fit statistic curves.^18^ The analysis was performed using the “flexmix” extension package in R (R Foundation for Statistical Computing).^19^

#### Hypothesis 2: correlates of hospitalization trajectory clusters

We used data from the index hospitalization to identify hospitalization and patient characteristics correlated with the patient's subsequent hospitalization trajectory. We fit logistic regression models in which we varied the response variable. For binary tests of cluster membership (eg, high vs low utilization trajectory), we fit binary logistic regression models. We also fit a proportional odds ordinal logistic regression in which all levels of the cluster variable were modeled as a single ordinal variable.^20^ All models were estimated using maximum likelihood estimation following multiple imputation of missing data via additive regression, bootstrapping, and predictive mean matching (10 imputed datasets) using the “rms” extension package in R.^21^

The study was approved by the VUMC Institutional Review Board. All data preparation and analysis were performed using the software packages Python (Python Software Foundation), R, and Stata (StataCorp).

## Results

### Sample description and summary statistics

Figure 1 shows the flow diagram for inclusion in the study. The sample was restricted to adult patients with complete hospital utilization episodes as defined above. In total, there were 3404 such patients, who collectively were hospitalized 11 183 times during the 24-month follow-up period of each episode. Together with hospitalizations occurring during the low-risk (2990 hospitalizations) and rising-risk (10 212 hospitalizations) periods for each patient, 88.9% of all hospitalizations for the 3404 patients were captured by the utilization episodes. The remaining 11.1% of hospitalizations were excluded because they occurred after the 24-month follow-up period.

Table 1, column 1, presents summary statistics for the 3404 patients with qualifying utilization episodes. Reflecting the heterogeneous nature of the HNHC population, the sample reflects a broad demographic cross-section, with an interquartile age range of 39.0–65.9 years, approximately equal proportions of men and women, and a diverse mix of racial, marital, and insurance statuses. With regard to hospitalization characteristics, most (71.1%) patients were admitted to a medicine service for the index hospitalization and most (75.8%) were discharged home. Reflecting the complexity of these patients, one-third (33.4%) died during the utilization episode.

**Table 1.** Summary statistics, full sample and by hospitalization trajectory cluster.

		Utilization trajectories			
	Full sample (*n* = 3404)	None (*n* = 523)	Low (*n* = 1406)	High-slow (*n* = 1035)	High-fast (*n* = 440)
	(1)	(2)	(3)	(4)	(5)
Patient characteristics					
** **Age, y					
Mean [IQR]	52.8 [39.0, 65.9]	51.5 [37.2, 64.6]	52.7 [39.0, 65.8]	53.6 [39.8, 66.3]	53.0 [39.4, 66.8]
Sex					
Male	1785 (52.4%)	267 (51.1%)	712 (50.6%)	577 (55.7%)	229 (52.0%)
Female	1619 (47.6%)	256 (48.9%)	694 (49.4%)	458 (44.3%)	211 (48.0%)
Race					
Black	768 (22.6%)	111 (21.2%)	313 (22.3%)	243 (23.5%)	101 (23.0%)
White	2495 (73.3%)	382 (73.0%)	1035 (73.6%)	754 (72.9%)	324 (73.6%)
Other or unknown	141 (4.1%)	30 (5.7%)	58 (4.1%)	38 (3.7%)	15 (3.4%)
Marital status					
Married	1283 (37.7%)	173 (33.1%)	498 (35.4%)	432 (41.7%)	180 (40.9%)
Divorced or separated	488 (14.3%)	77 (14.7%)	210 (14.9%)	138 (13.3%)	63 (14.3%)
Widowed	282 (8.3%)	42 (8.0%)	125 (8.9%)	77 (7.4%)	38 (8.6%)
Single	1341 (39.4%)	229 (43.8%)	567 (40.3%)	387 (37.4%)	158 (35.9%)
Unknown	10 (0.3%)	2 (0.4%)	6 (0.4%)	1 (0.1%)	1 (0.2%)
Insurance					
Commercial	737 (21.7%)	123 (23.5%)	283 (20.1%)	220 (21.3%)	111 (25.2%)
Medicare	1714 (50.4%)	224 (42.8%)	721 (51.3%)	549 (53.0%)	220 (50.0%)
Medicaid	595 (17.5%)	110 (21.0%)	253 (18.0%)	166 (16.0%)	66 (15.0%)
Uninsured	247 (7.3%)	53 (10.1%)	104 (7.4%)	65 (6.3%)	25 (5.7%)
Unknown	111 (3.3%)	13 (2.5%)	45 (3.2%)	35 (3.4%)	18 (4.1%)
Household income					
Below sample median	1614 (47.4%)	240 (45.9%)	657 (46.7%)	510 (49.3%)	207 (47.0%)
Above sample median	1774 (52.1%)	280 (53.5%)	741 (52.7%)	521 (50.3%)	232 (52.7%)
Unknown	16 (0.5%)	3 (0.6%)	8 (0.6%)	4 (0.4%)	1 (0.2%)
Has primary care provider					
No	839 (24.6%)	172 (32.9%)	352 (25.0%)	223 (21.5%)	92 (20.9%)
Yes	2565 (75.4%)	351 (67.1%)	1054 (75.0%)	812 (78.5%)	348 (79.1%)
Index hospitalization characteristics					
Length of stay, days					
Mean [IQR]	6.58 [2.68, 7.98]	6.90 [2.79, 8.27]	6.65 [2.65, 7.98]	6.73 [2.71, 8.05]	5.65 [2.60, 7.33]
Admission type					
Acute	2536 (74.5%)	373 (71.3%)	1055 (75.0%)	782 (75.6%)	326 (74.1%)
Elective	868 (25.5%)	150 (28.7%)	351 (25.0%)	253 (24.4%)	114 (25.9%)
Service					
Medicine	2421 (71.1%)	313 (59.8%)	950 (67.6%)	809 (78.2%)	349 (79.3%)
Psychiatry	497 (14.6%)	120 (22.9%)	229 (16.3%)	112 (10.8%)	36 (8.2%)
Surgery	426 (12.5%)	82 (15.7%)	200 (14.2%)	98 (9.5%)	46 (10.5%)
Other	60 (1.8%)	8 (1.5%)	27 (1.9%)	16 (1.5%)	9 (2.0%)
ICU-level care					
No	2884 (84.7%)	435 (83.2%)	1212 (86.2%)	865 (83.6%)	372 (84.5%)
Yes	520 (15.3%)	88 (16.8%)	194 (13.8%)	170 (16.4%)	68 (15.5%)
Code status					
Full code (confirmed)	1593 (46.8%)	167 (31.9%)	635 (45.2%)	544 (52.6%)	247 (56.1%)
Full code (presumed)	1501 (44.1%)	258 (49.3%)	654 (46.5%)	427 (41.3%)	162 (36.8%)
DNR or DNR/DNI	310 (9.1%)	98 (18.7%)	117 (8.3%)	64 (6.2%)	31 (7.0%)
Discharge location					
Facility	824 (24.2%)	167 (31.9%)	357 (25.4%)	217 (21.0%)	83 (18.9%)
Home	2580 (75.8%)	356 (68.1%)	1049 (74.6%)	818 (79.0%)	357 (81.1%)
Utilization episode characteristics					
Total hospitalizations					
Mean [IQR]	3.29 [1.00, 4.00]	0 [0, 0]	2.22 [1.00, 2.00]	5.70 [3.00, 7.00]	4.93 [3.00, 6.00]
Died during episode					
No	2266 (66.6%)	394 (75.3%)	1020 (72.5%)	592 (57.2%)	260 (59.1%)
Yes	1138 (33.4%)	129 (24.7%)	386 (27.5%)	443 (42.8%)	180 (40.9%)

“Household income” is the median household income in the patient's zip code of residence according to the 2021 American Community Survey (US Census Bureau).

Abbreviations: DNI, do not intubate; DNR, do not resuscitate; ICU, intensive care unit.

### Hospitalization trajectories

Figure S2 plots the hospitalization trajectories of all 3404 patients with qualifying utilization episodes. As stipulated by the case definition, before the index hospitalization, patients had a low-risk period in which there were no more than 2 hospitalizations per patient in a 6-month period. This was followed by a rising-risk period in which all patients had 3 hospitalizations in a maximum of 6 months. After the third (ie, index) hospitalization in this sequence, a wide dispersion in hospitalization trajectories was observed, ranging from no further hospitalizations over the following 24 months to a maximum of 38 hospitalizations.

### Hypothesis 1: identification of hospitalization trajectory clusters

**Figure 2.**
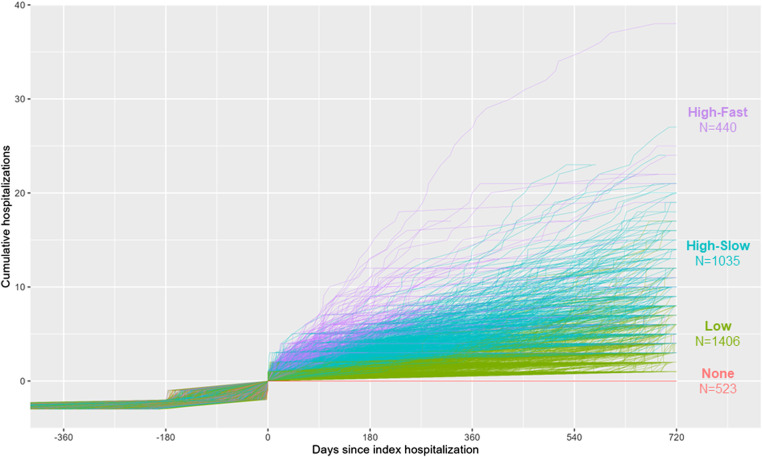
Individual hospitalization trajectories, by cluster (*n* = 3404). The “high-fast,” “high-slow,” and “low” hospitalization trajectory clusters were assigned by growth mixture modeling of a 3-cluster model of linear-quadratic trajectories. The “none” utilization trajectory was excluded from the mixture model due to non-convergence and then re-incorporated afterwards. Time is indexed to the index hospitalization—ie, the third hospitalization in the first instance of 3 hospitalizations in a 6-month period. Trajectories ending before 720 days after the index hospitalization indicate death.

Figure S3 plots fit statistics across the 5 growth mixture models that we tested, varying in cluster count from 1 to 5. We chose the 3-cluster model on the basis of the “elbow” in the fit statistic curves at this cluster count—ie, the point after which there was relative flattening of the fit statistic curves, indicating no further improvement in fit. For reasons detailed in the legend to Figure 2, the 3-cluster model resulted in 4 total clusters inclusive of those who had no hospitalizations after the index hospitalization.

Figure 2 plots the individual hospitalization trajectories grouped by cluster. On account of the shapes of the trajectories, we labeled them as a “no” utilization cluster (*n* = 523), a “low” utilization cluster (*n* = 1406), and 2 high utilization clusters—one with a slow rate of increase (“high-slow”; *n* = 1035) and the other with a fast rate of increase (“high-fast”; *n* = 440). Table 1, columns 2–5, show summary statistics for the 4 clusters. Mean (IQR) hospitalizations during the follow-up period were 0 (0–0), 2.2 (1–2), 5.7 (3–7), and 4.9 (3–6) for the none, low, high-slow, and high-fast clusters, respectively.

### Hypothesis 2: correlates of hospitalization trajectory clusters

Table 2 examines 3 sets of baseline variables as potential predictors of hospitalization trajectory cluster: characteristics of the index hospitalization, patient demographics, and patient comorbidities at the time of the index hospitalization. As an initial test of characterizing high vs low chronic utilization irrespective of trajectory/pace of utilization, we combined the 2 high-utilization trajectory clusters (high-fast and high-slow) and the 2 low-utilization trajectory clusters (none and low) for the model in column 1. Controlling for index hospitalization characteristics and patient comorbidities, none of the demographic variables was independently correlated with subsequent high vs low utilization. In contrast, several features of the index hospitalization—admission to a nonsurgical service, full code status, ICU-level care during the hospitalization, administration of opioid medications, and discharge home—were correlated with higher odds of subsequent high utilization. Moreover, several comorbidities present at the index hospitalization were correlated with high subsequent utilization—ie, major cardiovascular disease, end-stage kidney or liver disease, and cancer. In contrast, the presence of a psychiatric disorder was correlated with no or low subsequent utilization, and the presence of a substance use disorder, chronic pain diagnosis, and SDOH diagnosis were uncorrelated with subsequent utilization.

**Table 2.** Correlates of hospitalization trajectory clusters.

	(1)			(2)			(3)		
	High-fast + high-slow vs none + low			Ordinal			High-fast vs high-slow		
	aOR	95% CI	*P*	aOR	95% CI	*P*	aOR	95% CI	*P*
Index hospitalization									
Length of stay (days)	0.99	0.98-1.00	.126	0.99	0.98-1.00	.073	0.98	0.96-1.00	.**042**
Full code	2.23	1.70-2.91	**<**.**001**	2.72	2.14-3.46	**<**.**001**	0.84	0.53-1.33	.447
Acute admission	1.02	0.87-1.21	.792	1.09	0.94-1.26	.265	0.96	0.73-1.26	.786
Surgical service	0.57	0.46-0.72	**<**.**001**	0.62	0.51-0.76	**<**.**001**	1.14	0.77-1.67	.513
ICU-level care	1.23	1.00-1.52	.**045**	1.12	0.93-1.35	.219	1.08	0.77-1.51	.648
Any opioid medication administered	1.25	1.07-1.46	.**004**	1.19	1.04-1.36	.**012**	0.82	0.64-1.05	.115
Discharged to home	1.28	1.07-1.52	.**006**	1.27	1.08-1.48	.**003**	1.03	0.76-1.39	.864
Patient demographics									
Above median age	0.91	0.76-1.08	.258	0.90	0.77-1.05	.194	0.97	0.73-1.28	.832
Male sex	1.14	0.99-1.32	.071	1.08	0.95-1.23	.233	0.83	0.65-1.05	.122
Non-White race	1.03	0.87-1.21	.766	1.00	0.87-1.16	.964	1.00	0.77-1.31	.984
Married	1.12	0.95-1.31	.173	1.06	0.92-1.23	.395	0.85	0.66-1.11	.242
Above median income	0.93	0.81-1.08	.346	0.96	0.85-1.09	.516	1.08	0.86-1.37	.493
Medicare	1.07	0.88-1.29	.495	1.14	0.96-1.35	.143	0.84	0.62-1.14	.257
Medicaid or uninsured	0.91	0.73-1.14	.414	0.92	0.75-1.12	.388	0.81	0.56-1.18	.272
Has PCP	1.13	0.95-1.35	.170	1.18	1.01-1.37	.**042**	1.03	0.77-1.38	.862
Patient comorbidities									
Total chronic diagnoses	1.00	0.97-1.03	.999	1.00	0.97-1.03	.842	0.98	0.93-1.03	.468
Any major cardiovascular disease	1.23	1.03-1.46	.**020**	1.21	1.04-1.41	.**015**	0.91	0.69-1.21	.520
End-stage liver/kidney disease	1.30	1.08-1.56	.**005**	1.32	1.12-1.56	.**001**	1.36	1.03-1.80	.**030**
Any cancer	1.47	1.23-1.76	**<**.**001**	1.52	1.29-1.78	**<**.**001**	1.47	1.12-1.94	.**006**
Any substance use disorder	0.87	0.72-1.05	.136	0.86	0.73-1.02	.077	1.11	0.81-1.52	.506
Any psychiatric disorder	0.83	0.70-0.98	.**027**	0.81	0.70-0.95	.**007**	0.90	0.69-1.18	.451
Any chronic pain diagnosis	1.09	0.91-1.30	.348	1.02	0.87-1.20	.763	0.92	0.68-1.23	.559
Any SDOH diagnosis	1.14	0.84-1.57	.402	1.00	0.76-1.33	.977	1.07	0.63-1.82	.810
No.	3404			3404			1475		
Pseudo-*R^2^*	0.065			0.069			0.030		

Each column (1–3) reports the aORs of a single logistic regression of the column header on the row covariates, which are drawn from the index hospitalization. Columns 1 and 3 are binary logistic regressions. Column 2 is a proportional odds ordinal logistic regression in which all 4 hospitalization trajectory clusters have been modeled as an ordinal variable in the following ascending order: none, low, high-slow, and high-fast. All models were estimated using maximum likelihood estimation after multiple imputation of missing data via additive regression, bootstrapping, and predictive mean matching with 10 imputed datasets. The covariates used in the imputation model were the same row covariates as in the main regression models. Bold font indicates *P* < 0.05.

Abbreviations: aOR, adjusted odds ratio; ICU, intensive care unit; PCP, primary care provider; SDOH, social determinants of health.

To further investigate whether baseline characteristics were correlated with the pace in addition to the level of subsequent hospitalization, in column 2, we conducted an ordinal regression in which the response variable had 4 levels for each successive level of hospitalization trajectory: none, low, high-slow, and high-fast. The results were largely similar to the collapsed high vs low analysis, although the ordinal model provided slightly better fit (pseudo-*R^2^* of 0.069 vs 0.065).

Finally, to investigate whether baseline characteristics could differentiate fast vs slow utilization, we compared the high-fast vs high-slow hospitalization trajectories. Column 3 shows that a shorter length of stay during the index hospitalization was correlated with a faster pace of rehospitalization. The presence of cancer or end-stage kidney or liver disease was also significantly correlated with faster rehospitalization.

The heatmaps in Figures S4 and S5 provide further suggestive evidence of cluster-based differences in chronic comorbidities and medications administered during the index hospitalization. In particular, the prevalence of anemia and administration of intravenous fluids both increased monotonically across the 4 clusters.

## Discussion

This study aimed to identify distinct hospitalization trajectories and determine their correlates among adult patients at a large academic medical center. Our results build on prior research identifying discrete demographic subgroups within the HNHC population, and extend this research by finding that hospitalization trajectories are heterogeneous, clinically relevant, and correlated more strongly with hospitalization and clinical characteristics than demographic traits.

Specifically, our analysis revealed 4 unique hospitalization trajectories, including 2 that reflected contrasting paces of high utilization. Notably, it revealed that the majority (53.2%) of patients ultimately had no more than 2 further hospitalizations during the 2 years after their rising-risk period, highlighting the importance of the prediction task of differentiating those likely to become persistently rather than merely transiently high utilizers.

With regard to this prediction task, persistently high utilizers were more likely to be medically (as opposed to psychiatrically or socioeconomically) complex medical (as opposed to surgical) patients who were treatment-oriented (full-code) and independently living (discharged home). To illustrate, the data suggest that a typical “high and fast” utilizer may be a patient with a new cancer diagnosis undergoing active treatment and hospitalization for complications. On the other hand, a typical “high and slow” utilizer may be a patient with heart failure who has periodic hospitalizations for exacerbations. Finally, a prototypical low utilizer may be a patient with psychiatric disease but no significant medical comorbidities. That this picture contrasts with prior evidence indicating high psychiatric and substance use burden and low socioeconomic status as key risk factors for ED utilization^2,4^ may be due to different underlying processes driving ED vs inpatient utilization and/or methodological differences such as our study's focus on incident rather than prevalent high utilization.

Our study has several limitations. First, the results of this study are based on data from a single institution; as such, besides standard external validity concerns, internal validity may have been compromised if patients were frequently hospitalized at other institutions. However, we have previously found that the vast majority of patients ever admitted to VUMC are admitted exclusively to VUMC.^22^ Second, because our data were drawn exclusively from the EHR (as opposed to supplemented by additional assessments), we were subject to its documentation constraints. For example, we relied on ICD-10 “Z codes” to identify SDOH factors such as housing and food insecurity. To the extent these factors were under-coded relative to their true burden, our results regarding SDOH may have been biased towards the null. Finally, we examined hospitalizations only, but ED usage and length of stay are other important dimensions of hospital utilization that may have different predictive and preventive factors.

Despite these limitations, our study has 2 key methodological strengths. First, our use of EHR data is an outlier in the HNHC literature, which more commonly has drawn on insurance claims data to characterize the HNHC population.^2^ Our EHR-based dataset allowed us to query granular aspects of hospitalization (eg, code status, primary service) that were found to be highly correlated with subsequent utilization. Second, this study's conceptualization of utilization episodes allowed us to identify incident high utilization and make unbiased comparisons across patients. This methodology can be readily applied to other EHR-based datasets, especially those drawn from the widely used Epic platform—ie, the potential for scale-up of our research approach is high. Taken together, our study has considerable policy relevance, as the identification of patients who are most likely to become persistently high utilizers of hospital care is a critical issue for policymakers, health plans, and health systems, especially as they transition to value-based payment systems where readmission rates are a key quality and reimbursement metric. Likewise, given the disproportionate contribution of HNHC patients to health care costs, reducing unnecessary utilization in this population is increasingly a priority for capitated payment systems that assume financial risk for patient costs.

In summary, our study demonstrates that the emergence and evolution of high hospital utilization over time can be systematically studied using EHR data. In particular, longitudinal analysis provides insights into the heterogeneity of utilization trajectories and their prediction, thus laying the groundwork for early identification of patients most likely to have high utilization and become true HNHC patients.

## Supplementary material

Supplementary material is available at *Health Affairs Scholar* online.

## Supplementary Material

qxad077_Supplementary_Data
