# Supplementary figures and images for: Characterizing hospitalization trajectories in the high-need, high-cost population using electronic health record data
Source: Health Aff Sch. 2023 Dec 6;1(6):qxad077. doi: 10.1093/haschl/qxad077 (PMC10986247; doi:10.1093/haschl/qxad077)

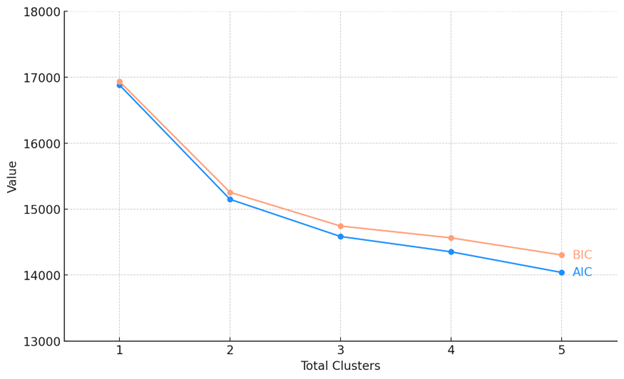

Supplement: qxad077_Supplementary_Data [file qxad077_Supplementary_Data.zip › Health_Affairs_Scholar_Supp_Figure_3_fit_statistics.tif]

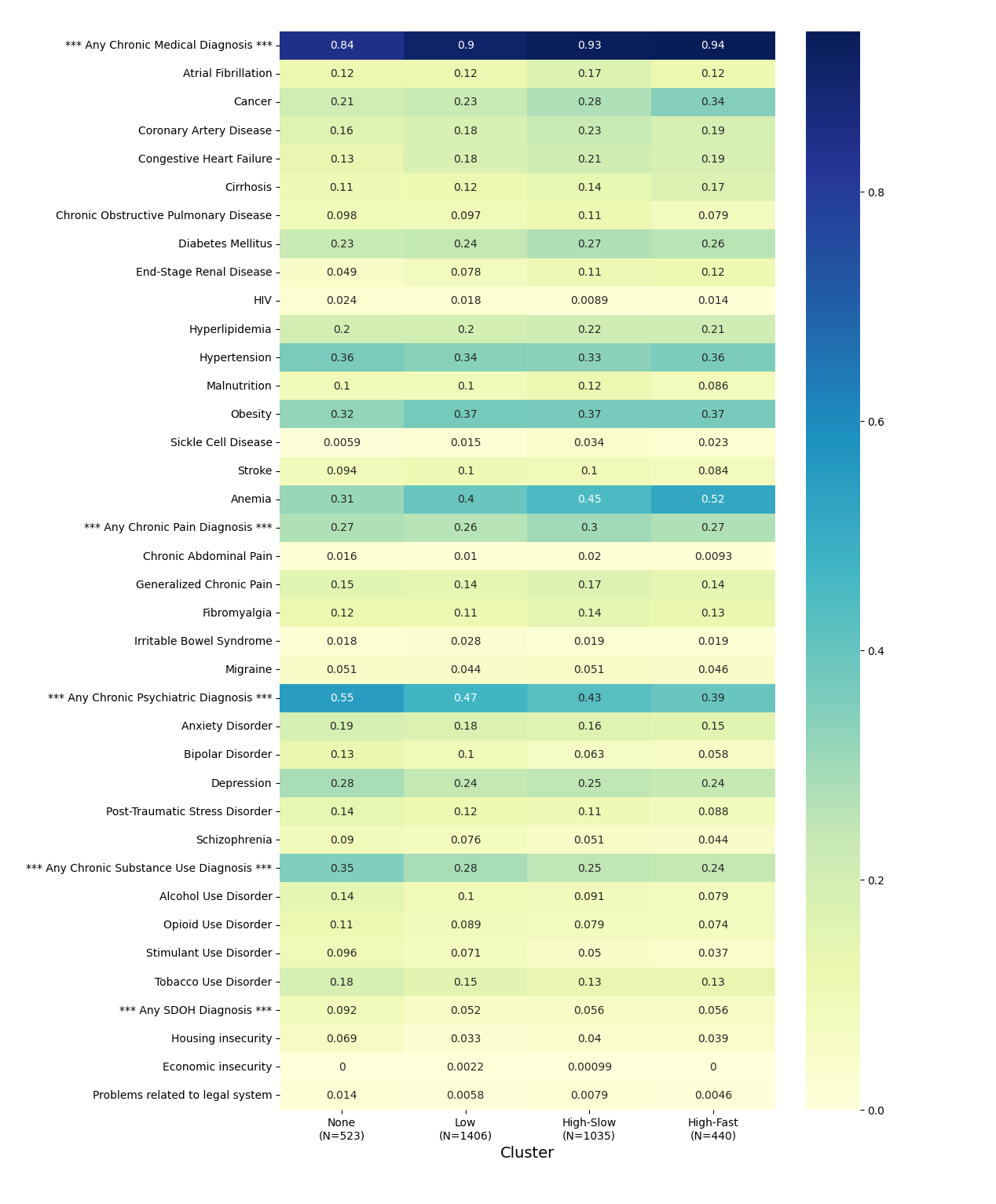

Supplement: qxad077_Supplementary_Data [file qxad077_Supplementary_Data.zip › Health_Affairs_Scholar_Supp_Figure_4_heatmap_dx_REVISED.tif]

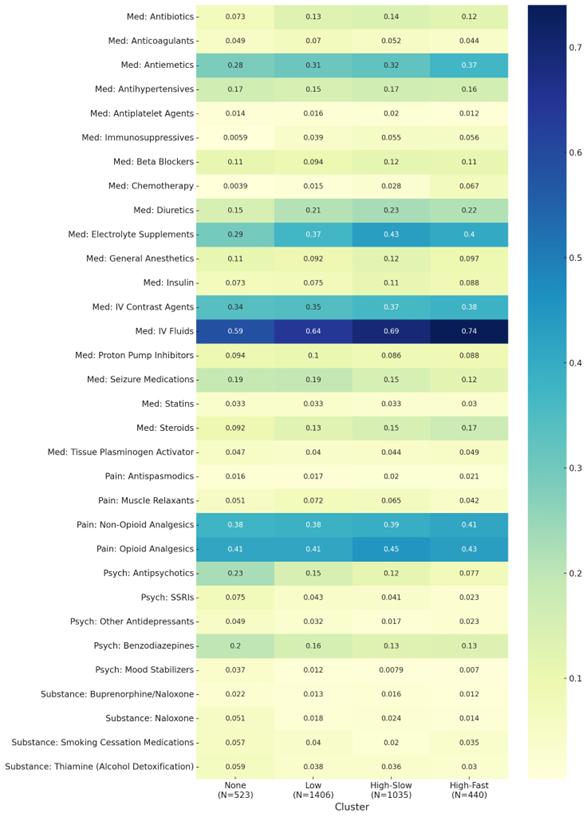

Supplement: qxad077_Supplementary_Data [file qxad077_Supplementary_Data.zip › Health_Affairs_Scholar_Supp_Figure_5_heatmap_med.tif]

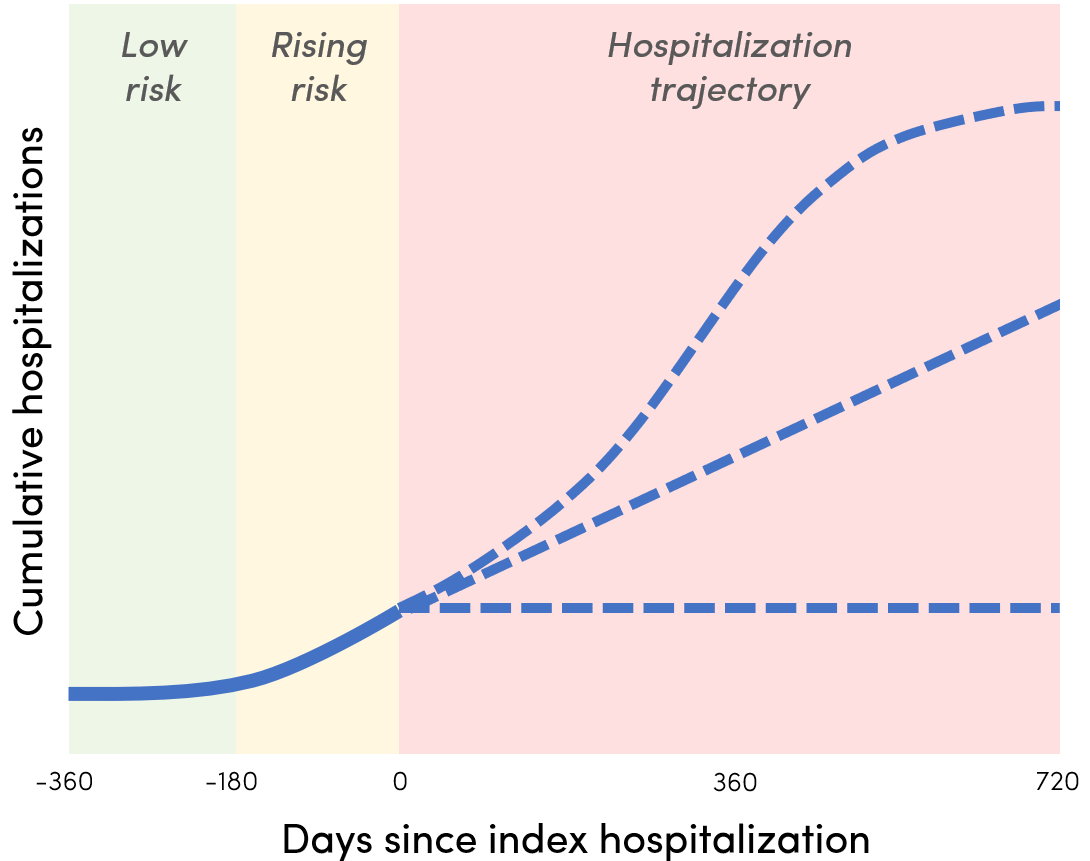

Supplement: qxad077_Supplementary_Data [file qxad077_Supplementary_Data.zip › Health_Affairs_Scholar_Supp_Figure_1_trajectories_concept.tif]

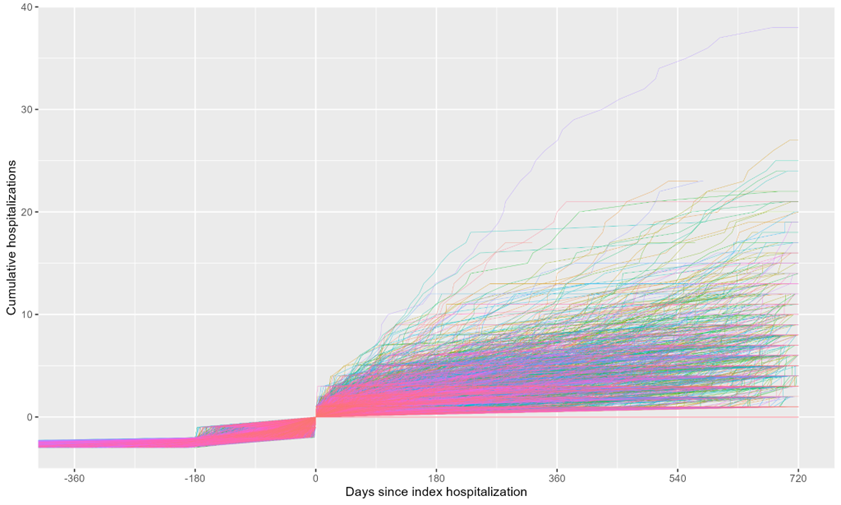

Supplement: qxad077_Supplementary_Data [file qxad077_Supplementary_Data.zip › Health_Affairs_Scholar_Supp_Figure_2_trajectories.tif]
